# A new endophyte *Monascus ruber* SRZ112 as an efficient production platform of natural pigments using agro-industrial wastes

**DOI:** 10.1038/s41598-022-16269-1

**Published:** 2022-07-23

**Authors:** El-Sayed R. El-Sayed, Joanna Gach, Teresa Olejniczak, Filip Boratyński

**Affiliations:** 1 Plant Research Department, Nuclear Research Center, Egyptian Atomic Energy Authority, Cairo, Egypt; 2 Department of Food Chemistry and Biocatalysis, Wrocław University of Environmental and Life Sciences, Norwida 25, 50-375 Wrocław, Poland

**Keywords:** Biotechnology, Microbiology

## Abstract

A number of biopigment applications in various industrial sectors are gaining importance due to the growing consumer interest in their natural origin. Thus, this work was conducted to valorize endophytic fungi as an efficient production platform for natural pigments. A promising strain isolated from leaves of *Origanum majorana* was identified as *Monascus ruber* SRZ112 produced several types of pigments. The nature of the pigments, mainly rubropunctamine, monascin, ankaflavin, rubropunctatin, and monascorubrin in the fungal extract was studied by LC/ESI–MS/MS analyses. As a first step towards developing an efficient production of red pigments, the suitability of seven types of agro-industrial waste was evaluated. The highest yield of red pigments was obtained using potato peel moistened with mineral salt broth as a culture medium. To increase yield of red pigments, favourable culture conditions including incubation temperature, incubation period, pH of moistening agent, inoculum concentration, substrate weight and moisture level were evaluated. Additionally, yield of red pigments was intensified after the exposure of *M. ruber* SRZ112 spores to 1.00 KGy gamma rays. The final yield was improved by a 22.12-fold increase from 23.55 to 3351.87 AU g^−1^. The anticancer and antioxidant properties of the pigment’s extract from the fungal culture were also studied. The obtained data indicated activity of the extract against human breast cancer cell lines with no significant cytotoxicity against normal cell lines. The extract also showed a free radical scavenging potential. This is the first report, to our knowledge, on the isolation of the endophytic *M. ruber* SRZ112 strain with the successful production of natural pigments under solid-state fermentation using potato peel as a substrate.

## Introduction

Natural pigments consist of several types of bioactive compounds used extensively in several applications of biopigments in different industrial sectors, as food colorants, dietary supplements, pharmaceuticals, cosmetics, aquaculture and poultry feed^1^. Currently, natural pigments in comparison to the synthetic colors have taken a lead in a rapidly changing industry. The number of synthetic colorants allowed by the regulatory agencies is quite dependent of the compound and application purpose. So, their number has decreased due to their potential carcinogenicity as well as their undesirable mutagenicity. The European Union has recommended in 2010 the acceptable levels of some colorings for consumption every day. Also, the World Health Organization and US Food and Drug Administration limited the usage of synthetic colors in drugs and food products^2^. Moreover, a chemical synthesis of pigments is a complex process and generates unwanted by-products and hazardous wastes affecting the environment^3^. Nowadays, extraction of natural pigments from plants and animals have gained more attention^4^; however, it is an expensive process with very low yield and environmentally costly^5^. By 2024, it was expected that the world color market used in food industry will reach approximately 5.7 billion US dollar^6^. Unfortunately, the basic science and industry still have not overcome the high cost and low availability of natural pigments^7^. Therefore, the demand for the naturally produced biopigments will continue to rise. Microbial pigments, among the natural pigments, showed several advantages including the ease of scaling up and harvest^8^ as well as not subjected to the nature vagaries^9^. Fungal platforms for production of biopigments are of great interest as environmentally safer substitutes for synthetic ones. Fungi, in comparison with algal and bacterial platforms, are more efficient biotechnology agents due to the high mycelial growth rate^10^ and fungal response to the metabolic regulators in large-scale production processes^8^. In addition, the approval by the EU in 2000 for the use of filamentous fungal pigments as food colorants (such as hydroxyanthraquinoid pigments) has opened up a new era for the use of fungi as pigments producers^2^.

Recently, fungal endophytes i.e. fungi colonizing the inside of healthy plant tissues, have gained immense attention because they are a lucrative source of several bioactive compounds^11–14^. In the literature, information regarding strategies to discover new fungal strains and maximizing their production at industrial scale are lacking. So, attention is still focused on unexplored habitats such as plants for isolation of new and hyper-producing strains (endophytic fungi), which is the important step toward the design of biotechnological processes for pigments production^8^. Besides, agro-industrial wastes could be used as low-cost substrates for the pigment-production by fungi^1^ in solid-state fermentation (SSF) technology, since synthetic culture media are usually expensive^15^. SSF is the process of microbial cultivation in the complete or near absence of free water^16^, thereby resembling the natural habitat of the fungus. It enhances the microbial resistance to catabolic repression^17^, intensifies the microbial growth, and induces the production of metabolites to elevated levels^18^. As such, improvement of pigment producing microbial strains for obtaining hyper-producers not being harmful for the consumer could be performed using physical mutagens^19^ such as gamma irradiation^20,21^. For all of these reasons, we aim to unfold the untapped potential of endophytic fungi as efficient producers of red pigments. Different agro-industrial wastes, namely potato peel, wheat bran, rice bran, sugarcane bagasse, corn cobs, wheat husk, and rice husk, were tested for their suitability as substrates for production of red pigments. Moreover, the effect of exposure to gamma rays on the red pigment-overexpressing by the fungal strain was studied.

## Materials and methods

### Isolation and identification of the pigments producing fungus

Collection of plant materials used for endophyte isolation in this study was performed according to the institutional, national, and international guidelines and legislation. The fungus was isolated from healthy leaves of the plant *Origanum majorana* according to method of the fungal endophytes’ isolation^22^. The isolated cultures were checked for purity and stored in glycerol (15%, w/v) as a suspension of spores and mycelia at − 4 °C.

Identification of the fungal strain was accomplished by colony morphology, growth characteristics, and molecular characterization. Morphological identification was performed by studying the colony on Czapek's-yeast autolysate agar according to universal keys^23,24^. Molecular characterization was performed using PCR-amplified ITS1-5.8S-ITS2 rRNA-gene^25^. In brief, DNA of the fungal strain was extracted and sequenced by Solgent Company, Daejeon, South Korea. Sequences of the strain was submitted to the GenBank and accession numbers were received. Finally, sequences of the fungal strain were analyzed using the online tool BLAST (http://www.ncbi.nlm.nih.gov/) and the software BioEdit (version 7.0.1). A neighbor-joining tree with the maximum-likelihood for the fungal strain were constructed using MEGA software version 6.0.

The pigments-producing fungal strain was finally determined as *Monascus ruber* SRZ112 (GenBank accession number MT140350.1) and deposited (deposition number AUMC14390) in the culture collection of Assiut University Mycological Center (http://www.aun.edu.eg/aumc/aumc.htm), Assiut, Egypt.

### Collection and preparation of agro-industrial wastes

Potato peel (was collected from domestic wastes), wheat bran, rice bran (was purchased from local stores), sugarcane bagasse (was obtained from a sugarcane mill), corn cobs, wheat husk, and rice husk (was collected from cultivated area) were screened in this study for their potentiality as cultural substrates for red pigments production by *Monascus ruber* under solid-state fermentation (SSF). The waste materials were kept in sterile plastic bags and transported to the laboratory. All the waste materials were dried in a hot-air oven-dried at 45 °C. All the waste materials were milled, sieved (to an average particle size of 0.2–0.5 mm), and finally stored at 7 °C.

### Production of pigments by *M. ruber* SRZ112

Agar slants (7 days-old) of *M. ruber* SRZ112 were flooded by a sterile saline solution then gently scrapping-off spores and finally vortexed for 1 min. To adjust the spore concentration to 10^6^ spore/mL, the collected spores were diluted and counted using a hemocytometer. Production of pigments were achieved using solid-state cultivation as follows; in a 250 mL Erlenmeyer flask 10 g potato peel (substrate) was moistened by 6 mL of the Mineral Salt Broth (pH 6.0) to attain a moisture content of 60%, w/w and autoclaved. After cooling to room temperature, 1 mL of the prepared spore suspension was added to the flask and the contents were thoroughly mixed. The flasks were dark-incubated for 6 days at 25 °C and the pigments were extracted, as described later.

### Red pigments production under SSF

Red amongst the identified pigments was selected for yield-improving program via SSF by the *M. ruber* SRZ112 strain because of its high industrial interest^19^. All the collected wastes were screened for their suitability as cultural substrates for red pigment production. In addition, two moistening agents were tested for maximum production of red pigments. To each flask, 10 g of the tested substrate was moistened by 6 mL of either distilled water (DW) or mineral-salt broth (MSB) to attain a moisture content of 60%, (w/w). Flasks were autoclaved, cooled, and separately inoculated by 1 mL of the prepared spore suspension. The flasks were dark-incubated for 6 days at 25 °C.

### Effect of different fermentation conditions on production of red pigment

The effect of different fermentation variables on red pigment production by the *M. ruber* SRZ112 grown on potato peel moistened by MSB (the most favorable for red pigment production) were assessed by One Factor at a Time design. These variables were different incubation temperatures (20–40 °C), effective incubation period by incubating the flasks for 20 days, initial pH of the MSB (adjusted using 1 N NaOH or 1 N HCl in the range 3.0–8.0), inoculum concentration (10^4^–10^8^ spore/mL), substrate weight per 250-mL flask (5–30 g/flask), and moisture level (50–90%, w/w).

### Effect of ^60^Co gamma-irradiation on growth and red pigment production

Spore suspension of the *Monascus ruber* SRZ112 was prepared and irradiated by gamma rays in the range 0.25–4.00 KGy using a ^60^Co Gamma chamber (Model: MC20, Russia). Immediately following irradiation, suspensions were stored in darkness (to avoid photoreactivation) at 4 °C overnight. After which, the irradiated suspensions were separately tested for red pigment production using 15 g potato peel moistened by MSB at 70% moisture level, incubated at 30 °C for 10 days, the most favorable conditions for red pigment production from the previous experiment.

### Analytical methods

#### Estimation of the fungal growth

Growth of the *Monascus ruber* SRZ112 was estimated by quantifying the released N-acetyl glucosamine from the acid-hydrolyzed chitin in the fungal cell wall^26^. Glucosamine concentrations were expressed as mg g^−1^ dry fermented substrate and estimated after recording the UV absorption at 530 nm from a standard curve^16^.

#### Extraction of red pigment

Red pigment was extracted according to a procedure reported previously with slight modifications^27^. At the end of the incubation period, 10 mL of absolute ethanol was added to 1 g of fermented substrate. Then, the mixture was incubated for 4 h with an agitation speed of 200 rpm. After that, the mixture was filtered through Whatman paper No 1 and further centrifuged at 45×*g* for 10 min. The supernatant was diluted and used for estimation of the red pigments yield.

#### Determination of the red pigment yield

Red pigment production was evaluated by spectrophotometric measurements (JENWAY-305, UK) at 500 nm in terms of absorbance units per g dry fermented substrate (AU g^−1^) according to a previously reported method^28^, taking into consideration the dilution factor of the sample^29^. Red pigment yield was estimated and expressed as absorbance units which were multiplied by the dilution factor^30^.

#### Chemical identification of pigments by LC/ESI–MS/MS analysis

The crude pigment extract of the gamma irradiated *M. ruber* SRZ112 was subjected to LC/ESI–MS/MS analysis to identify the present chemical constituents. LC/ESI–MS/MS measurements were performed by a RSLC Dionex UltiMate 3000 (Thermo Fisher Scientfic, USA) with mass spectrometer ESI-Q-TOF, maXis impact (Bruker, USA) with Syncronis Phenyl C18 1.7 µm, 100 × 2.1 mm, column (Thermo Scientific, USA). The mobile phase was a mixture of 0.5% aqueous formic acid v/v (A) and 0.5% formic acid in acetonitrile (B), the flow rate was 0.2 mL/min, column incubation temperature was 30 °C. The program was: 0 min—80% A, 20% B; 0–10 min—50% A, 50% B; 10.0–14.4 min—15% A, 85% B; 14.4–14.8 min—5%A, 95% B; 14.8–16.0 min 80% A, 20% B; 16.0–24.0 min 80% A, 20% B. The major operating parameters were: nebulizer pressure: 2.0 bar, heating gas flow: 8.0 L/min, heating gas temperature: 220 °C, data acquisition range: m/z 50–1500 Da, ionization mode: positive, ion source energy: 15 eV.

### Determination of the anticancer activity

In order to evaluate the cytotoxicity of the crude pigment extract, the ethanol extracts were pooled, evaporated and dissolved in 0.1% DMSO to obtain a desired range of concentrations. Cytotoxicity of the pigments extracted from SSF culture of *M. ruber* SRZ112 was evaluated against normal human melanocytes (HFB-4) and human breast carcinoma (MCF-7) by the MTT-based assay^31^. In brief, both normal human melanocytes and human breast carcinoma cells were treated separately with 20 μL of the prepared crude pigment extract. After-which, the MTT solution was added to the wells, and the absorbance was recorded at 570 nm using a microplate reader (SunRise, TECAN, Inc., USA). Wells containing cells treated with 0.1% DMSO (without pigments) served as controls. The relative viability of the treated cells was estimated by the following equation:

$${\text{Viability }}\left( \% \right) = \left( {{\text{Absorbance of treated cells}}/{\text{ Absorbance of control cells}}} \right)\, \times \,100.$$

The IC_50_ values, 50% inhibitory concentration, were calculated using GraphPad Prism software, San Diego, CA.

### Determination of the antioxidant activity

In order to evaluate the antioxidant potential of the crude pigment extract, the ethanol extracts were pooled, evaporated and dissolved in methanol to obtain a desired range of concentrations. Antioxidant potential of the crude pigments extracted from SSF culture of *M ruber* SRZ112 was estimated by the free radical scavenging assay^32^ using 2,2-diphenyl picrylhydrazyl (DPPH, Sigma-Aldrich, St. Louis, MO, USA). Simultaneously, a positive control of ascorbic acid (Sigma-Aldrich, St. Louis, MO, USA) was also tested at the same concentrations. Scavenging activity (%) was calculated as the change in the absorbance of the mixture (DPPH + pigments) with respect to the DPPH solution only (control).

### Statistical analysis

Calculated mean is for triplicate measurements from two independent experiments. Statistical significance of the obtained means was analyzed by the analysis of variance (One-Way ANOVA) and the Least Significant Difference (LSD) tests (at 0.05 level) using SPSS software version 22 (IBM Corp).

## Results

### Isolation and identification of the fungal strain

**Figure 1:**
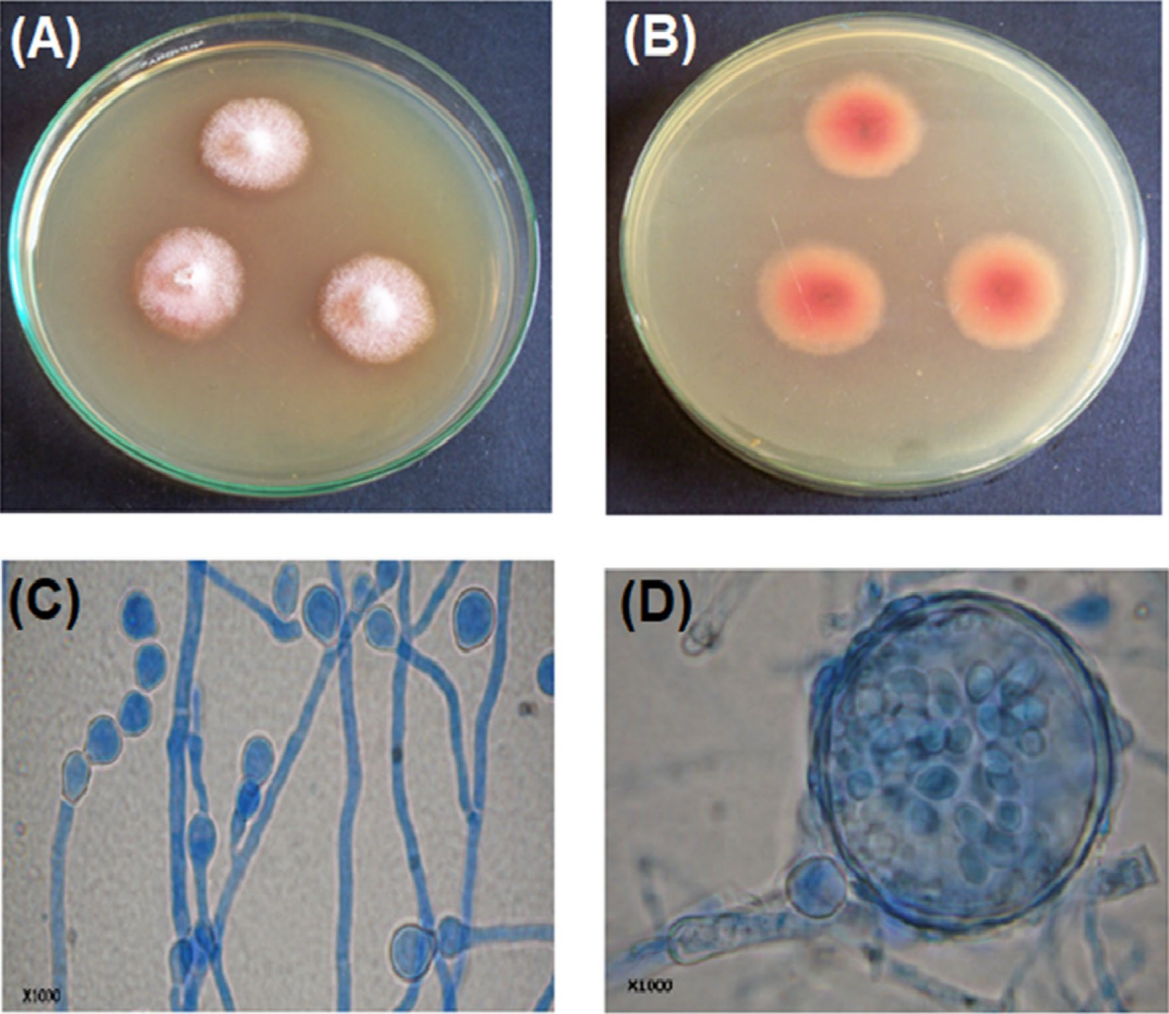
Morphological characteristics of the red pigments-producing fungal strain. Colony growth was observed on Czapek–Yeast autolystae agar. The plate cultures on the left show a front view of the growth (**A**), and the plate cultures on the right show a reverse view (**B**) of the growth after incubation for 10 days at 25 °C. Appearance under light microscope (**C**,**D**).

**Figure 2:**
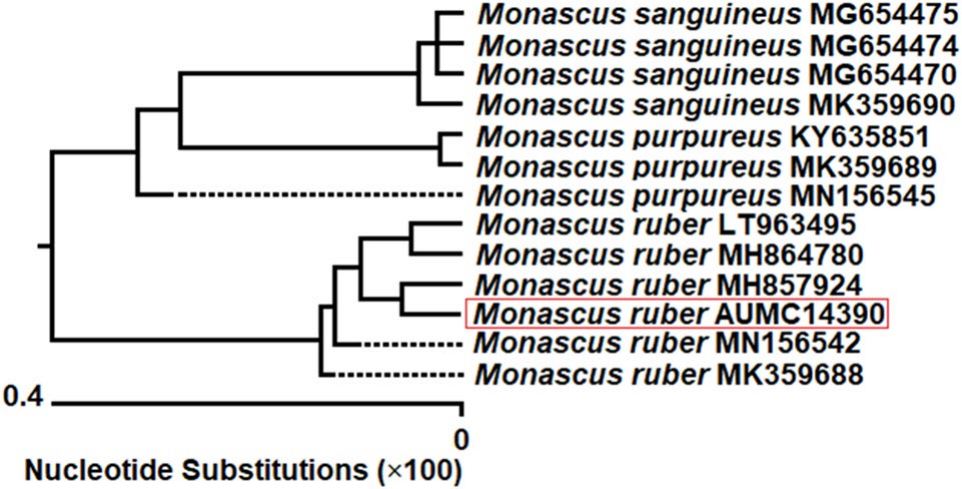
Phylogenetic tree of the pigment-producing fungal strain (AUMC14390) and other closely related strains of *Monascus*, based on the ITS1-5.8S rRNA-ITS2 rDNA sequences.

The pigment producing fungal strain namely SRZ112 was isolated from healthy leaves of the medicinal herb *Origanum majorana*. Figure 1 presented morphological features with growing colonies of the SRZ112 strain on Czapek's-yeast autolysate agar plates. After 10 days of growth at 25 °C, red pigmentation was observed in both front (Fig. 1A) and reverse (Fig. 1B) view of the growing colonies. In addition, chains of a sexual conidia, young ascoma (Fig. 1C), and the mature ascoma with ellipsoidal ascospores (Fig. 1D) were observed under microscope. Identification of the SRZ112 strain was confirmed by amplification and sequencing of 18S rRNA of the SRZ112 strain. The retrieved sequences were deposited (under accession number MT140350.1) in the GenBank. Using BLAST tools, the retrieved sequences and those of the closely related species accessed from the GenBank were compared. Figure 2 presented the constructed phylogenetic tree indicating that the SRZ112 strain had a 100% identity with *Monascus ruber* strains.

### Suitability of agro-industrial wastes for maximum red pigment production

Data presented in Table 1 showed that growth of *M. ruber* SRZ112 and red pigments yield were greatly influenced by the used waste and the type of moistening agent. Among the tested wastes, potato peel moistened by MSB was the best solid substrate. The recorded values of growth and red pigment concentration were 10.21 ± 0.32 mg g^−1^ and 23.55 ± 0.83 AU g^−1^, representing the highest yield of red pigments. Moreover, rice bran followed by sugarcane bagasse were also good substrates for red pigment production by *M. ruber* SRZ112. On using rice bran moistened by MSB, values of the growth and red pigment were 8.66 ± 0.22 mg g^−1^ and 20.64 ± 0.42 AU g^−1^. Also, these values were 7.32 ± 0.39 mg g^−1^ and 17.78 ± 0.21 AU g^−1^, on using sugarcane bagasse. However, rice husk, corn cobs, and wheat husk were very poor substrate for either the growth of *M. ruber* SRZ112 or production of red pigment (Table 1).

**Table 1:** Fungal growth (mg glucosamine g^−1^ dry fermented substrate) and red pigment yield (AU g^−1^ dry fermented substrate) of *M. ruber* SRZ112 grown on potato peel, rice bran, wheat bran, sugarcane bagasse, rice husk, wheat husk, and corn cobs moistened with two moistening agents.

Solid substrate	Moistening agent	Glucosamine concentration (mg g^−1^)	Red pigment yield (AU g^−1^)
Potato peel	MSB	10.21 ± 0.32^a^	23.55 ± 0.83^a^
	DW	4.82 ± 0.15^b^	10.65 ± 0.02^c^
Rice bran	MSB	8.66 ± 0.22^a^	20.64 ± 0.42^a^
	DW	6.87 ± 0.51^a^	8.54 ± 0.11^de^
Rice husk	MSB	1.43 ± 0.08^c^	5.31 ± 0.64^e^
	DW	0.92 ± 0.07^b^	3.98 ± 0.21^f^
Sugarcane bagasse	MSB	7.32 ± 0.39^a^	17.78 ± 0.21^b^
	DW	4.33 ± 0.82^bc^	10.45 ± 0.00^c^
Corn cobs	MSB	2.76 ± 0.45^c^	6.23 ± 0.12^de^
	DW	0.52 ± 0.03^b^	2.45 ± 0.52^e^
Wheat bran	MSB	6.43 ± 0.87^a^	11.98 ± 0.43^c^
	DW	5.01 ± 0.34^a^	6.89 ± 0.76^de^
Wheat husk	MSB	0.94 ± 0.02^c^	4.73 ± 0.09^de^
	DW	0.76 ± 0.01^c^	1.89 ± 0.89^e^

Mineral salt broth (MSB) was composed of (g L^−1^) MgSO_4_ × 7H_2_O 0.5, KCl 0.5, and FeSO_4_ × 7H_2_O 0.01.

Initial pH of all moistening agents was adjusted to 6.0 using 1 N NaOH and HCl. Solid–state grown cultures were carried out at 25 °C for 6 days. Calculated mean is for triplicate measurements from two independent experiments ± SD, ^a–e^means with different superscripts in the same column for each individual substrate are considered statistically different (LSD test, *P* ≤ 0.05).

### Effect of different cultivation conditions on production of red pigment

**Figure 3:**
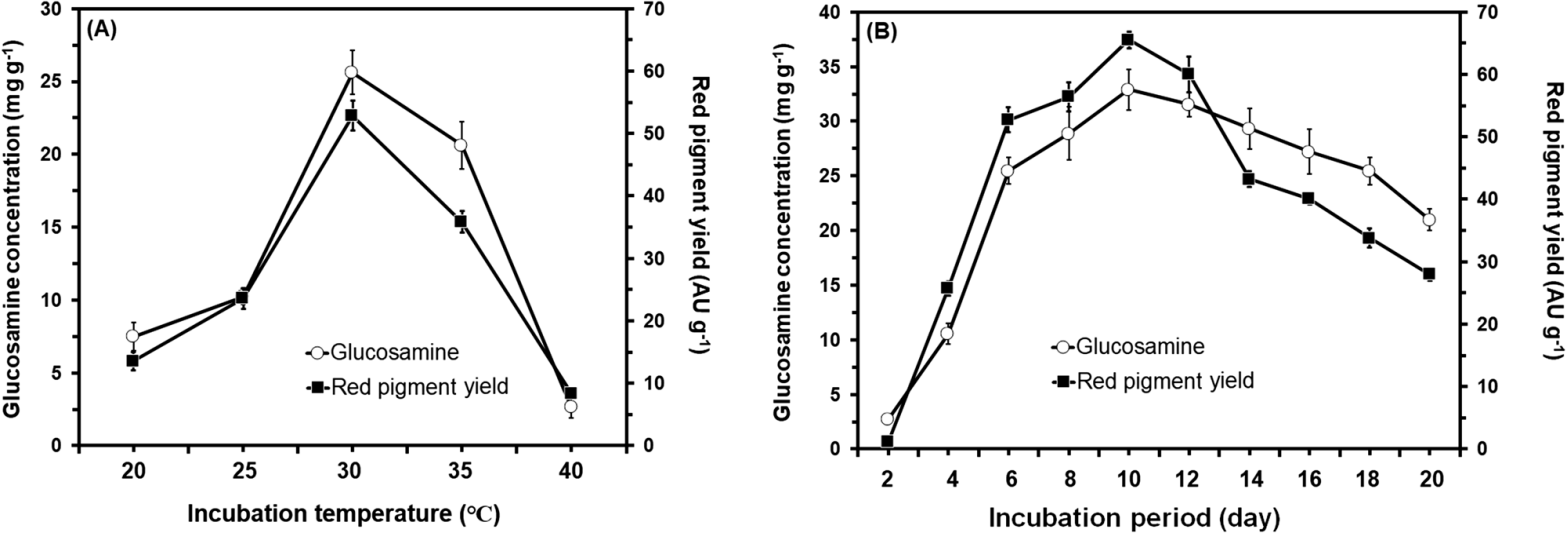
Effect of different incubation temperatures (**A**) and effect of different incubation periods (**B**) on growth (mg g^−1^) and red pigments production (AU g^−1^) by *Monascus ruber* SRZ112 under SSF. Initial pH of the MSB moistening agent was adjusted to 6.0 using 1 N NaOH and HCl. Solid–state grown cultures were carried out on potato peel. All data are shown as the mean ± SD of triplicate measurements from two independent experiments.

**Figure 4:**
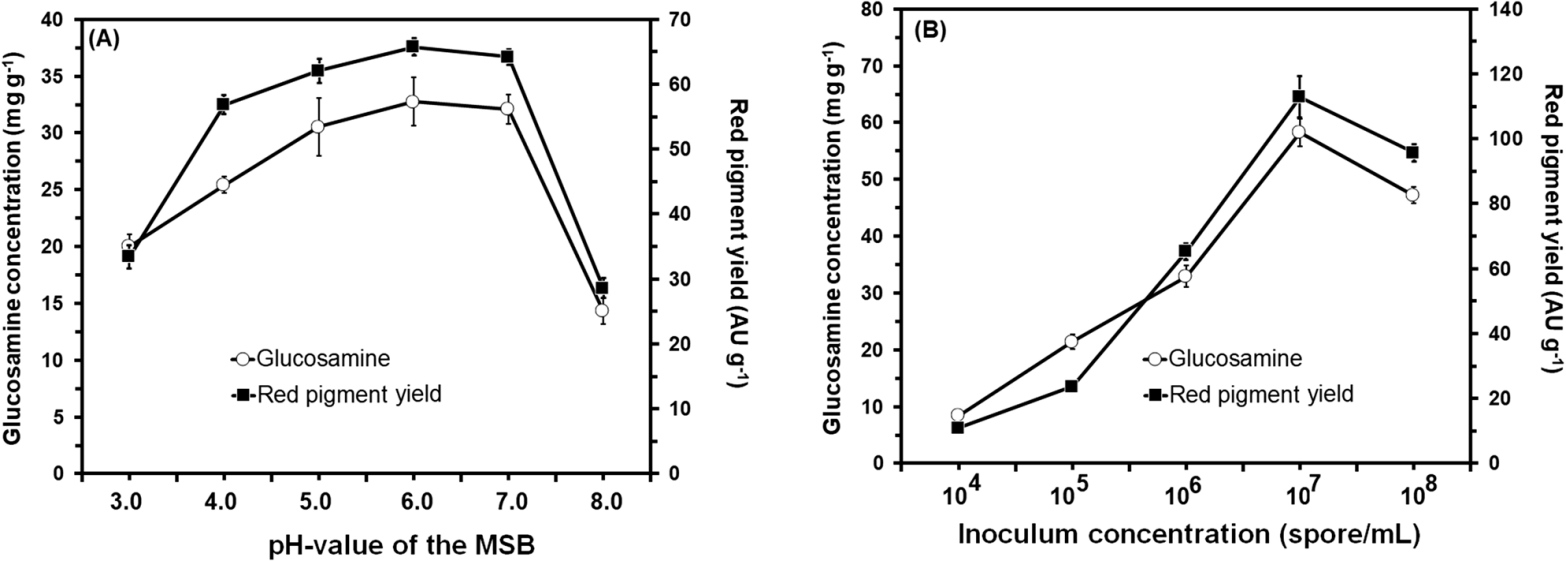
Effect of adjusting the pH of the MSB to different values (**A**) and effect of different inoculum concentrations (**B**) on growth (mg g^−1^) and red pigments production (AU g^−1^) by *Monascus ruber* SRZ112 under SSF. Initial pH of the MSB moistening agent was adjusted to varying values using 1 N NaOH and HCl. Solid–state grown cultures were carried out on potato peel at 30 °C for 10 days. All data are shown as the mean ± SD of triplicate measurements from two independent experiments.

**Figure 5:**
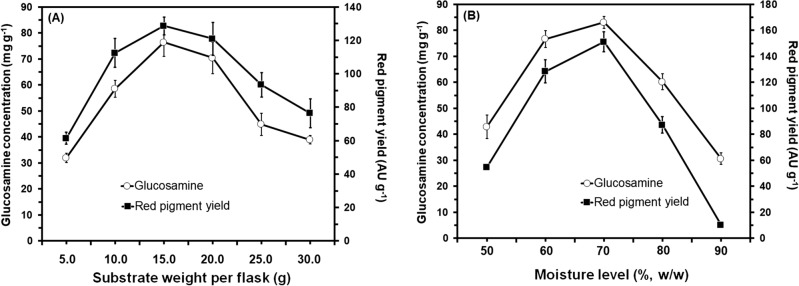
Effect of different weights of potato peel as substrate **(A)** and effect of different moisture levels **(B)** on growth (mg g^−1^) and red pigments production (AU g^−1^) by *Monascus ruber* SRZ112 under SSF. Initial pH of the MSB moistening agent was adjusted to 6.0 using 1 N NaOH and HCl. Solid–state grown cultures were carried out on potato peel at 30 °C for 10 days. All data are shown as the mean ± SD of triplicate measurements from two independent experiments.

Generally, the experimental results indicated that both of the growth and red pigments production by *M. ruber* SRZ112 were greatly affected by changing the cultivation conditions (Figs. 3, 4, and 5). It was noticed that there was a significant increase (P ≤ 0.05) in growth of *M. ruber* SRZ112 and production of red pigment at all the tested conditions. Among the tested incubation temperatures 30 °C was the most proper for maximum yield of red pigment where a significant yield enhancement (P ≤ 0.05) was obtained. Conversely, fermentation that was conducted either at the higher temperature extreme (40 °C) or at lower ones (20 °C) reduced the obtained red pigment yield from *M. ruber* SRZ112 (Fig. 3A). Optimum incubation period for maximum red pigment production was recorded after 10 days (Fig. 3B) at using moistening agent with pH value adjusted at 6.0 (Fig. 4A). The most favorable inoculum concentration for red pigments yield was observed at 10^7^ spore/mL (Fig. 4B). Changing substrate weight (potato peel) dramatically affected the red pigment production specially at a 15 g where maximum yield was attained (Fig. 5A). The highest yield of red pigments was achieved on using potato peel moistened by MSB at 70%, w/w moisture level (Fig. 5B).

### Production enhancement of red pigment using ^60^Co gamma irradiation

Exposure of the* M. ruber* SRZ112 spores to several doses of gamma rays was used to study their effects on the production of red pigments. Results illustrated in Table 2 clearly confirmed the gamma rays’ dose-related manner effect on the growth of *M. ruber* SRZ112. It was also noticed from Table 2 that the highest reduction in the growth was recorded after receiving higher doses at 2.00 KGy. In addition, the* M. ruber* SRZ112 spores failed to grow after exposure to 4.00 KGy. However, cultures from spores receiving 1.00 KGy promoted the highest red pigment yield. Significant differences (P ≤ 0.05) in the recorded values of the red pigment yield were found at this dose and the yield reached 3351.87 ± 12.45 AU g^−1^. Table 2 further indicated that receiving higher dose of gamma-irradiation at 2.00 KGy resulted in a decline in the red pigment.

**Table 2:** Survival rate (%), fungal growth (mg glucosamine g^−1^ dry fermented substrate) and red pigment production (AU g^−1^ dry fermented substrate) of *M. ruber* SRZ112 grown under SSF at different doses of gamma irradiation.

Gamma-irradiation dose (Gy)	Survival rate (%)	Glucosamine concentration (mg g^−1^)	Red pigment yield (AU g^−1^)
0.0 (C)	100	84.21 ± 8.78^a^	151.55 ± 10.41^d^
0.25	91	71.65 ± 5.21^b^	776.32 ± 14.21^c^
0.50	76	65.82 ± 4.31^c^	1245.21 ± 14.29^b^
1.00	48	55.31 ± 2.54^d^	3351.87 ± 12.45^a^
2.00	4	7.21 ± 0.54^e^	87.21 ± 5.21^e^
4.00	0.0	0.00 ± 0.00^f^	0.00 ± 0.00^f^

Solid–state grown cultures were grown on potato peel moistened with MSB (pH 6.0) incubated at 30 °C for 10 days. Calculated mean is for triplicate measurements from two independent experiments ± SD, ^a–f^means with different superscripts in the same column either for carbon or nitrogen sources are considered statistically different (LSD test, *P* ≤ 0.05).

### Chemical identification of pigments produced by *M. ruber* SRZ112

**Figure 6:**
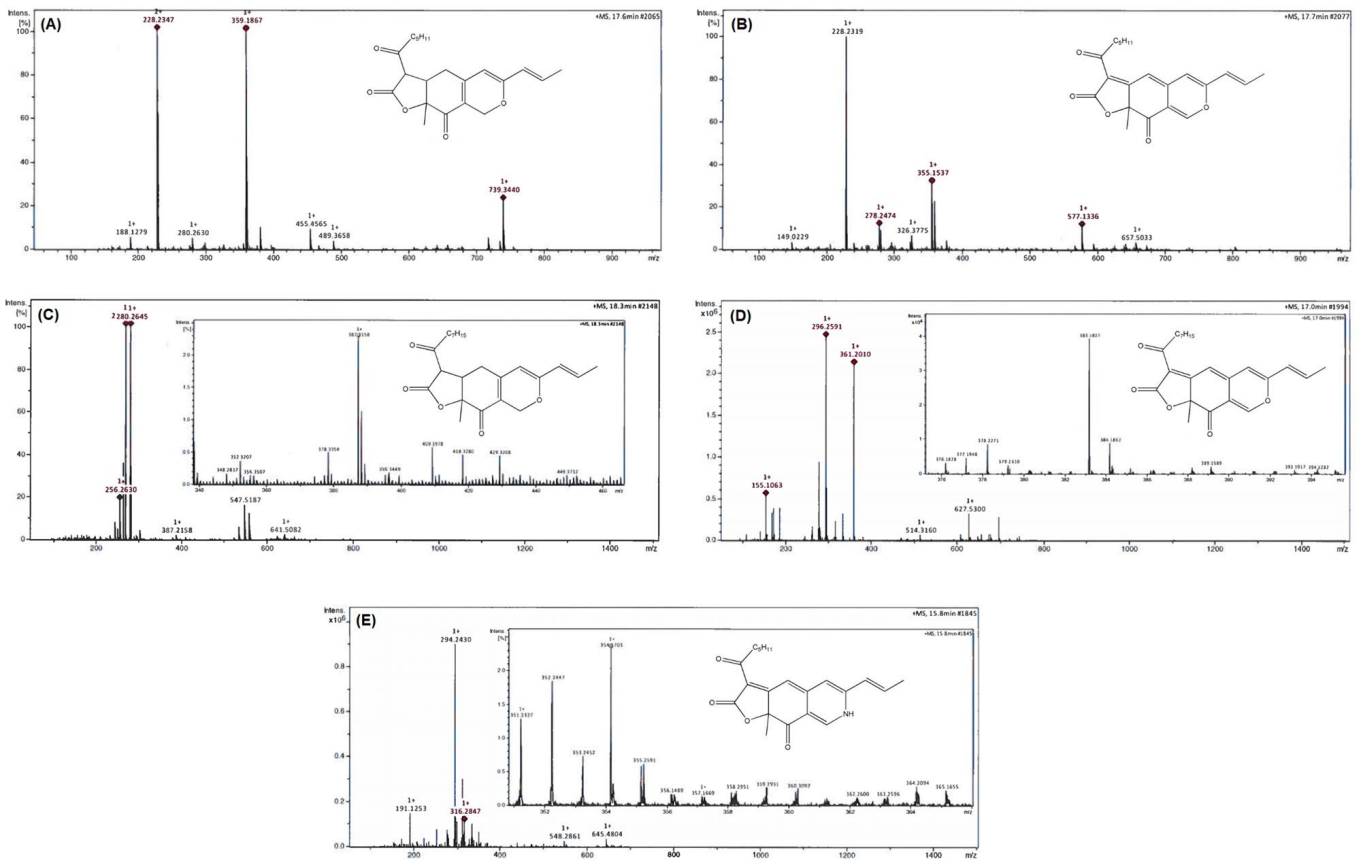
LC/ESI–MS/MS chromatograms of pigments extracted from SSF cultures of the gamma irradiated *M. ruber* SRZ112. Spectrum and structure of monascin (**A**). Spectrum and structure of rubropunctatin (**B**). Spectrum and structure of ankaflavin (**C**). Spectrum and structure of monascorubrin (**D**). Spectrum and structure of rubropunctamine (**E**).

Based on the LC/ESI–MS/MS spectra (Fig. 6), five different compounds were determined as well as their names and structures were also assigned. From the obtained data, the recorded [M+H]^+^ values were 359.1867 for monascin (Fig. 6A), 355.1537 for rubropunctatin (Fig. 6B), 387.2158 for ankaflavin (Fig. 6C), 383.1847 for monascorubrin (Fig. 6D), and 354.1703 for rubropunctamine (Fig. 6E). Moreover, no fragments corresponding to the mycotoxin citrinin were detected.

### Anticancer and antioxidant properties of pigments extracted from *M. ruber* SRZ112 culture

Table 3 showed that pigments extracted from cultures of the endophyte *M. ruber* SRZ112 grown on potato peel were active against the malignant MCF-7 breast cancer cell lines meanwhile no significant cytotoxic activity towards normal human melanocytes (HFB-4). The least inhibitory concentration of pigments extract against MCF-7 cell lines was 10 µg mL^−1^. Data also showed that the increase in pigment concentration resulted in a significant decrease in MCF-7 cell proliferation indicating the concentration-dependent cell death. Data in Table 3 also showed that the estimated IC_50_ value of the pigment extract against the MCF-7 cell lines was 64.32 µg mL^−1^.

**Table 3:** Cytotoxic and antioxidant activities of the crude pigment extracted from SSF cultures of *M. ruber* SRZ112 grown on potato peel.

Concentration (µg mL^−1^)	Cell viability (%)		Free radical scavenging activity (%)	
	MCF-7 (breast)	Hfb-4 (normal)	Pigments extract	Ascorbic acid
0.00 (C)	100.00 ± 0.00^a^	100.00 ± 0.00^a^	0.00 ± 0.00^d^	0.00 ± 0.00^d^
5	100.00 ± 0.00^a^	100.00 ± 0.00^a^	0.00 ± 0.00^d^	0.00 ± 0.00^d^
10	80.41 ± 3.21^b^	100.00 ± 0.00^a^	8.87 ± 2.65^c^	12.87 ± 2.76^c^
100	46.73 ± 9.42^c^	100.00 ± 0.00^a^	38.87 ± 5.05^b^	56.87 ± 8.61^b^
1000	12.41 ± 4.94^d^	98.43 ± 2.82^a^	75.48 ± 9.41^a^	100 ± 0.00^a^
IC_50_ (µg mL^−1^)	64.32	0.00	211.51	72.08

Calculated mean is for triplicate measurements from two independent experiments ± SD, ^a–d^Means with different superscripts in the same column are considered statistically different (LSD test, *P* ≤ 0.05).

Testing the antioxidant potential of pigments extracted from cultures of the endophyte *M. ruber* SRZ112 grown on potato peel clearly confirmed their antioxidant activity (Table 3) when compared by ascorbic acid at all the tested different concentrations. The recorded data confirmed the concentration-dependent manner of pigments extract in inhibition of free radicals. Data also revealed that the least inhibitory concentration of both ascorbic acid and pigment extract was 10 µg mL^−1^. Table 3 showed that the estimated IC_50_ values of pigment extract and ascorbic acid were 211.51 and 72.08 µg mL^−1^, respectively.

## Discussion

Nature provides several sources of pigments, despite that the high cost of their extraction and processing limits their availability, problems that industry still has not overcome. Thus, there are pressing scientific and social needs to promote the use of natural pigments as a safer and greener alternative for synthetic colors to reduce their health risks. Moreover, biopigments has challenged all the biotechnologists to search, characterize, develop a cost-effective and eco-friendly strategies for their production^19^. In this study, an endophytic fungus SRZ112 was isolated and found to produce pigments. The studied morphological characteristics of the SRZ112 were identical with universal keys^23,24^, concerning the identification of *Monascus ruber*. Also, molecular studies of the sequence from the SRZ112 strain revealed a 100% identity with those of *M. ruber*. In the literature, the are no data on the isolation of any endophytic *Monascus ruber* strains. As such, information and experience on using endophytic fungi as sources for production of pigments are very scarce^33,34^. Nevertheless, our study is the first report, to our knowledge, on the isolation of *M. ruber* from plant tissues. Generally, over 20 species of *Monascus* were reported; however, only few *M. ruber* strains have been recognized^35–37^.

Microbial pigments not only add color, but they also have interesting biological properties such as antioxidant^38^, antimicrobial^39^, anticancer, anti-inflammatory^19^, antiproliferative, and immunosuppressive activities^40^. Because of these pharmacological properties, this study was extended to evaluate the cytotoxic and antioxidant properties of pigments extracted from the culture of *M. ruber* SRZ112. Our results confirmed the antioxidant effect of pigments extracted from the fungal cultures. Moreover, our results indicated that pigments extracted from cultures of the endophyte *M. ruber* grown on potato peel were active against the malignant MCF-7 breast cancer cell lines meanwhile no significant cytotoxic activity towards normal human melanocytes (HFB-4). The same selective behavior was previously reported against normal diploid fibroblast cells^41^ and normal gastric epithelial cells^42^. Meanwhile, both of the pigment-rich fractions and the whole extract of the commercial Chinese red fermented rice showed inhibitory activities against colon cancer^43,44^.

Based on the promising anticancer and antioxidant properties of the pigments extract of *M. ruber* SRZ112, chemical constituents were investigated by LC/ESI–MS/MS analysis. Our results confirmed the presence of the following pigments; rubropunctatin (orange) monascin (yellow), ankaflavin (yellow), monascorubrin (orange), and rubropunctamine (red). The obtained data were in agreement with previous reports concerning the chemical characterization of the five pigments^21,44,45^. Moreover, no fragments corresponding to the mycotoxin citrinin were detected confirming that the produced culture extract is citrinin-free. Among these pigments, the red is of specific interest due to its potential for several therapeutic applications including antioxidant, immunosuppressive, antimicrobial^21^, anti-tumor, and cytotoxic potentials^45^. As such, red is the most common color used in food industry^19^. Thus, this study was directed to improve the red pigment yield by the *M. ruber* SRZ112 strain. As a first step, several agro-industrial residues were evaluated as cultural substrates for the production of red pigment by the endophytic fungus *M. ruber* SRZ112 grown under solid state fermentation. Over the years, SSF proved to be the most proper environment for a fungal growth^46^ and production of elevated rates of several metabolites of industrial significance application^47^. In particular, the possibility of using wastes generated by food and industry^48^ as well as the lower energy demand turned SSF into an economically viable and eco-friendly technology^49,50^. In this study, our results revealed that a noticeable variation in the obtained yield of red pigment was recorded according to the tested waste and moistening agent. Potato peel moistened by the MSB promoted the highest yield by *M. ruber* SRZ112. In the literature, a wide range of substrates were used for production of red pigments by several *Monascus* strains^19^. However, our study is the first report, to our knowledge, on using potato peel as a culture substrate for production of red pigments by *M. ruber* SRZ112. According to Liang and McDonald, the main components of potato peel waste (on dry basis) were 30% non-starch polysaccharide, 25% starch, 18% protein, 20% acid-soluble and acid-insoluble lignin, 1% lipids, and 6% ash^51^. Generally, the specific nature of the cultural substrate and composition of the moistening agent during a SSF had great influences on the process of red pigment production, as supported by several reports (^44^, and references therein).

Results of testing the effect of different cultivation conditions on the production of red pigment by *M. ruber* SRZ112 confirmed that the SSF process was greatly affected by changing the cultivation conditions as there is a significant increase in growth and red pigments yield. Of all the tested conditions, fermentation conducted at 30 °C for 10 days using moistening agent with pH value adjusted at 6.0 inoculated with inoculum concentration of 10^7^ spore/mL with 15 g potato peel moistened by MSB at 70%, w/w moisture level promoted the highest yields of red pigments. *Monascus* sp. is of a mesophilic nature which need moderate incubation temperature for optimal growth and maximum production rates^52^. Moreover, temperature between 30 and 37 °C was the most proper range for several *Monascus* isolates^27^. Conversely, high temperatures over 35 °C in rare cases were found the optimum for higher rates of pigment production^53^. In accordance with our results, Pawanjot stated that 10 days of incubation was the optimum for the production of *Monascus* pigments^54^. The author further reported a reduction in the pigment yield due to contamination. Similarly, the yield of pigment was increased with the incubation period; however, the prolonged incubation resulted in contaminations, depletion of medium ingredients, so the yield was reduced^55^. In partial agreement with our results, the favorable pH for red pigment production was at 5.5–8.5^56^. Moreover, higher pH (over 6.0) promoted synthesis of red pigment whereas lower pH (below 3.5) promoted synthesis of yellow pigments^45^. In agreement with our results, the highest pigment yield was achieved using an inoculum concentration between 10^6^ and 10^7^ spores/mL^54^. Moreover, the pigment productivity was decreased at elevated inoculum concentration due to the production of excess biomass^27^ and depletion of nutrients essential for pigment synthesis^55^. In agreement with our results, previous reports confirmed that moisture level and substrate weight greatly control the aeration of the SSF process^27,55^. Low-level moisture level adversely affects the metabolic activity by reducing nutrients solubility. In addition, the lower moisture can cause low degree of swelling of the substrate particles increasing the water tension and transfer of oxygen to the system^48,49^.

Herein, red pigment yield from *M. ruber* SRZ112 was significantly intensified after exposure to 1.00 KGy gamma rays. The obtained red pigment yield at this dose was 22.12 times higher than that obtained from the non-irradiated cultures. Exposure to mutagenic agents is a traditional method for stain improvement^21^. It mainly uses chemical, physical, and biological mutagens to induce mutations in the microbial genes, which can artificially guide biosynthesis of metabolic pathways and accumulate the desired metabolic product. For example, a mutant strain of *M*. *purpureus* designed as M630 with high pigments production was induced by exposure to room temperature plasma^57^. Generally, exposure to physical mutagens such as gamma irradiation^58^ and UV light^59,60^ may have a stimulatory effect on production of red pigment *M. ruber* SRZ112. Results obtained in this study also confirmed the negative effect of gamma-irradiation on the growth of *M. ruber* SRZ112. In accordance with these results, previous studies reported the same observation on the reducing effect of gamma radiation on the fungal growth^61,62^. In the literature, gamma rays as a mutagenic agent was highly recommended several reports^61,62^ for improvement of several fungal strains over-expressing a wide array of metabolites of industrial significance^63,64^. It is worthy here to mention that, several *Monascus* strains were reported in the literature with divers’ productivities. For instance, Babitha and co-workers achieved 12.113 OD Units/gds by *M. purpureus* LPB 97^27^. Zhang and co-workers reported the yield of 6.63 U/g by *M*. *purpureus* AS3.531^65^. Carvalho and co-workers used *Monascus* for pigment production 216 AU/g dry substrates^53^. Sharmila and co-workers achieved a pigment yield of 7.18 ODU/mL^66^. Embaby and co-workers obtained a maximal value of 108.02 ODU/ml by *M*. *purpureus* ATCC16436^30^. Pawanjot produced natural red pigment using *M*. *purpureus* a color value of 85 ODU/g dm^54^.

In summary, a new *Monascus* strain with pigment producing ability was isolated from plant tissues and identified to our knowledge for the first time. The fungal extract showed promising anticancer and antioxidant potentials. Chemical characterizations of pigments produced by the fungal strain were investigated. For an efficient red pigment solid-state fermentation, several wastes were screened where potato peel promoted the highest red pigment productivity. The favorable cultural conditions for maximum production of red pigments were studied. Furthermore, red pigment yield by *M. ruber* SRZ112 was intensified to a 22.12-fold increase from the initial titers after exposure to gamma rays.

## Data Availability

All data generated or analyzed during this study are included in this published article.
